# Supplementary material for: An evaluation of Chile’s Law of Food Labeling and Advertising on sugar-sweetened beverage purchases from 2015 to 2017: A before-and-after study
Source: PLoS Med. 2020 Feb 11;17(2):e1003015. doi: 10.1371/journal.pmed.1003015 (PMC7012389; doi:10.1371/journal.pmed.1003015)
Supplement: S1 Table — (DOCX) [file pmed.1003015.s001.docx]

**S1 Table. Nutrient thresholds and implementation dates
of the Chilean Labeling and Advertising Law**

| **Solid food** | **26 June 2016** | **26 June 2018** | **26 June 2019** |
| --- | --- | --- | --- |
| **Energy** (kcal/100g) | 350 | 300 | 275 |
| **Sodium** (mg/100g) | 800 | 500 | 400 |
| **Total sugars** (g/100g) | 22.5 | 15 | 10 |
| **Saturated fats** (g/100g) | 6 | 5 | 4 |
| **Liquids** | **26 June 2016** | **26 June 2018** | **26 June 2019** |
| **Energy** (kcal/100g) | 100 | 80 | 70 |
| **Sodium** (mg/100g) | 100 | 100 | 100 |
| **Total sugars** (g/100g) | 6 | 5 | 5 |
| **Saturated fats** (g/100g) | 3 | 3 | 3 |
