# Supplementary material for: An evaluation of Chile’s Law of Food Labeling and Advertising on sugar-sweetened beverage purchases from 2015 to 2017: A before-and-after study
Source: PLoS Med. 2020 Feb 11;17(2):e1003015. doi: 10.1371/journal.pmed.1003015 (PMC7012389; doi:10.1371/journal.pmed.1003015)
Supplement: S2 Table — (DOCX) [file pmed.1003015.s002.docx]

**S2 Table. Beverage groupings**

| **Sub-category** | **High-in^1^** | **Not high-in^2^** |
| --- | --- | --- |
| **Soda** | Carbonated, with added sugar | Carbonated, no added sugar or total sugar below  [**6 g/100 mL**] |
| **Fruit drinks** | Industrialized fruit-flavored drinks,  with added sugar | Industrialized fruit-flavored drinks, with no added sugar or total sugar below [**6 g/100 mL**] |
| **Dairy products** | Flavored milk powders, flavored  ready-to-drink milk, and flavored dairy | Plain milk, plain milk powders,  and plain dairy substitutes |
| **Waters** | Flavored powders and ready-to-drink  waters, with added sugar | Flavored powders, no added sugar or total sugar below [**6 g/100 mL**]; mineral water, sparkling water |
| **Coffee and tea** | n/a | Instant coffee, roasted coffee |
| **100% fruit and vegetable Juice** | n/a | 100% fruit and vegetable juices |
| **Sports and  Energy drinks** | Powdered and ready-to-drink sports drinks with added sugar, energy drinks with added sugar | Powdered and ready-to-drink sports drinks and  energy drinks, no added sugar or total sugar below  [**6 g/100 mL**] |

^1^ High-in beverages are those subject to the Chilean Law of Labeling and Advertising due to containing added sugars, saturated fats,
or salt and exceeding nutrient or energy thresholds.

^2^ Not high-in beverages are not subject to the Chilean Law of Labeling and Advertising because they either do not contain added sugars, saturated fats, or salt or they do contain one or more of those added ingredients but do not exceed nutrient or energy thresholds.
