# Supplementary material for: An evaluation of Chile’s Law of Food Labeling and Advertising on sugar-sweetened beverage purchases from 2015 to 2017: A before-and-after study
Source: PLoS Med. 2020 Feb 11;17(2):e1003015. doi: 10.1371/journal.pmed.1003015 (PMC7012389; doi:10.1371/journal.pmed.1003015)
Supplement: S3 Table — (DOCX) [file pmed.1003015.s003.docx]

**S3 Table. 12-month and 18-month analysis**

|  | **12-month window**^1^ | | **18-month window**^2^ | |
| --- | --- | --- | --- | --- |
|  | **Absolute** (95% CI) | **Relative %** (95% CI) | **Absolute** (95% CI) | **Relative %** (95% CI) |
| **Without month or seasonality adjustment** | -21.0 (-21.1, -20.9) | -21.7 (-21.7, -21.6) | -22.7 (-22.7, -22.5) | -23.6 (-23.7, -23.6) |
| **Adjusting for  quarter dummies** | -68.9 (-69.2, -68.6) | -47.9 (-47.9, -47.9) | -23.3 (-23.4, -23.2) | -24.2 (-24.2, -24.1) |
| **Month as continuous** | -24.3 (-24.4, -24.2) | -24.1 (-24.2, -24.1) | -22.5 (-22.6, -22.4) | -23.5 (-23.5, -23.4) |
| **Month as dummy** | 26.4 (26.4, 26.6) | - 1. (56.6, 56.9) | -22.7 (-22.8, -22.6) | -23.7 (-23.7, -23.6) |

^1^ 12 month window includes data 12 months before/after law implementation. The pre-period is defined as July 1, 2015 to June 30, 2016, and the post-period is defined as July 1, 2016 to June 30, 2017.

^2^ 18 month window includes data 18 months before/after law implementation. The pre-period is defined as January 1, 2015 to June 30^th^, 2016, and the post-period is defined as July 1, 2016 to December 31, 2017
