# Supplementary material for: An evaluation of Chile’s Law of Food Labeling and Advertising on sugar-sweetened beverage purchases from 2015 to 2017: A before-and-after study
Source: PLoS Med. 2020 Feb 11;17(2):e1003015. doi: 10.1371/journal.pmed.1003015 (PMC7012389; doi:10.1371/journal.pmed.1003015)
Supplement: S4 Table — (DOCX) [file pmed.1003015.s004.docx]

**S4 Table. Unadjusted percent of consumers^1^ who purchased^2^ high-in^3^ and not high-in^4^ beverages, overall and by beverage type, pre- and post-regulation**

|  | **Pre-regulation** | **Post-regulation** |  |  |
| --- | --- | --- | --- | --- |
|  | **% consumer^1^**  *(95% CI)* | **% consumer^1^**  *(95% CI)* | **Difference** | ***p*-value** |
| **High-in^3^** | 92.9  *(92.3, 93.5)* | 82.6  *(81.6, 83.7)* | -10.2  *(-11.4, -9.6)* | <0.001 |
| **Soda** | 81.4  *(80.3, 82.5)* | 77.7  *(76.4, 79.0)* | -3.7  *(-4.5, -2.8)* | <0.001 |
| **Fruit Drinks** | 47.2  *(45.9, 48.4)* | 4.2  *(3.8, 4.7)* | -42.9  *(-44.2, -41.7)* | <0.001 |
| **Waters** | 5.9  *(5.4, 6.3)* | 1.3  *(1.1, 1.5)* | -4.6  *(-5.0, -4.1)* | <0.001 |
| **Dairy** | 52.1  *(50.7, 53.5)* | 23.3  *(22.0, 24.5)* | -28.8  *(-30.2, -27.4)* | <0.001 |
| **Coffee** | — | — | — | — |
| **Not high-in^4^** | 96.6  *(96.2, 97.0)* | 97.6  *(97.3, 97.9)* | 1.0  *(0.7, 1.4)* | <0.001 |
| **Soda** | 34.4  *(32.9, 35.8)* | 38.1  *(36.6, 39.6)* | 3.7  *(2.6, 4.9)* | <0.001 |
| **Fruit Drinks** | 24.0  *(23.1, 25.0)* | 59.5  *(58.1, 60.8)* | 35.4  *(34.2, 36.7)* | <0.001 |
| **Waters** | 73.3  *(72.2, 74.4)* | 71.8  *(70.7, 73.0)* | -1.5  *(-2.6, -0.4)* | <0.005 |
| **Dairy** | 79.2  *(78.2, 80.3)* | 84.0  *(83.1, 85.0)* | 4.8  *(3.8, 5.8)* | <0.001 |
| **Coffee** | 48.9  *(47.5, 50.3)* | 47.9  *(46.5, 49.3)* | -1.0  *(-2.1, -0.2)* | 0.09 |

^1^ Percent of households that purchased >0 milliliters of that beverage type in a given month.

^2^ Purchase data provided by Kantar WorldPanel Chile.

^3^ High-in beverages are those subject to the Chilean Law of Labeling and Advertising due to containing added sugars, saturated fats, or salt and exceeding nutrient or energy thresholds.

^4^ Not high-in beverages are not subject to the Chilean Law of Labeling and Advertising because they either do not contain added sugars, saturated fats, or salt or they do contain one or more of those added ingredients but do not exceed nutrient or energy thresholds.
