# Supplementary material for: An evaluation of Chile’s Law of Food Labeling and Advertising on sugar-sweetened beverage purchases from 2015 to 2017: A before-and-after study
Source: PLoS Med. 2020 Feb 11;17(2):e1003015. doi: 10.1371/journal.pmed.1003015 (PMC7012389; doi:10.1371/journal.pmed.1003015)
Supplement: S6 Table — (DOCX) [file pmed.1003015.s006.docx]

**S6. Table Coefficient estimates from the models to estimate changes in purchases^1^
of high-in,^2^ not high-in,^3^ and total beverages**

| **Outcome variable** | **Pre-regulation trend**  **Coeff [SE]** | **Pre-post-regulation dummy**  **Coeff [SE]** | **Interaction between pre/post regulation dummy and count variable (time)**  **Coeff [SE]** |
| --- | --- | --- | --- |
| Logarithm mL/capita/day of  **high-in beverages** purchased | -0.007 [0.001]** | -0.181 [0.033]** | -0.002 [0.001] |
| Logarithm mL/capita/day of  **not high-in beverages** purchased | -0.002 [0.001] | 0.148 [0.041]** | -0.002 [0.001] |
| Logarithm mL/capita/day of  **total beverages** purchased | - 0.004 [0.001]** | -0.030 [0.032] | -0.005 [0.001] |

^1^ Purchase data provided by Kantar WorldPanel Chile.

^2^ High-in beverages are those subject to the Chilean Law of Labeling and Advertising due to containing added sugars, saturated fats, or salt and exceeding nutrient or energy thresholds.

^3^ Not high-in beverages are not subject to the Chilean Law of Labeling and Advertising because they either do not contain added sugars, saturated fats, or salt or they do contain one or more of those added ingredients but do not exceed nutrient or energy thresholds.

** p value<0.01
