# Supplementary material for: An evaluation of Chile’s Law of Food Labeling and Advertising on sugar-sweetened beverage purchases from 2015 to 2017: A before-and-after study
Source: PLoS Med. 2020 Feb 11;17(2):e1003015. doi: 10.1371/journal.pmed.1003015 (PMC7012389; doi:10.1371/journal.pmed.1003015)
Supplement: S7 Table — (DOCX) [file pmed.1003015.s007.docx]

**S7 Table. Coefficients for the fully interacted model^1^ with education level to estimate changes in purchases^2^ of high in beverages**

| **Variable** | **Coefficient** [95% confidence interval] |
| --- | --- |
| Post-regulation | -0.185 [-0.250,-0.120]** |
| Month Year | -0.007 [-0.009,-0.005]** |
| Post-regulation × month year | -0.001 [-0.004,0.002] |
| Post-regulation × month/year/completed high school^3^ | 0.0004 [-0.01,0.002] |
| Post-regulation × month/year/college or greater^4^ | -0.003 [-0.005,-0.001]** |

^1^ Adjusted for seasonality, household size and composition, wealth, unemployment and interactions of each variable with education. Reference group for education: less than high school.

^2^ Purchase data provided by Kantar WorldPanel Chile.

^3^ F test for Post-regulation*month/year/completed high school
F (1,2362) =0.77, Prob>F=0.3816

^4^ F test for Post-regulation*month/year/college or greater
F (1,2362) =8.77, Prob>F=0.031

***p*-value<0.001.
