# Supplementary material for: An evaluation of Chile’s Law of Food Labeling and Advertising on sugar-sweetened beverage purchases from 2015 to 2017: A before-and-after study
Source: PLoS Med. 2020 Feb 11;17(2):e1003015. doi: 10.1371/journal.pmed.1003015 (PMC7012389; doi:10.1371/journal.pmed.1003015)
Supplement: S8 Table — (DOCX) [file pmed.1003015.s008.docx]

**S8 Table. Model testing**

|  | **Fixed effects, 2-part model, logged outcome, Duan smearing factors applied** | **Fixed effects, 2-part model, logged outcome, no Duan smearing factors applied** | **Generalized Estimating Equation, 2-part-model, log link** |
| --- | --- | --- | --- |
| **High-in^1^ beverages** |  |  |  |
| **Absolute difference** (mL/capita/day) | -22.8 | -25.1 | -33.6 |
| **Relative difference** (%) | -23.7 | -31.8 | -30.7 |
| **Not high-in^2^ beverages^3^** |  |  |  |
| **Absolute difference**  (mL/capita/day) | 14.6 | 17.1 | -5.2 |
| **Relative difference (%)** | 4.8 | 9.6 | -1.8 |

Results represent the mean absolute and relative difference between the observed beverage purchases and counterfactual beverage purchases in the post-regulation period.

^1^ High-in beverages are those subject to the Chilean Law of Labeling and Advertising due to containing added sugars, saturated fats, or salt and exceeding nutrient or energy thresholds.

^2^ Not high-in beverages are not subject to the Chilean Law of Labeling and Advertising because they either do not contain added sugars, saturated fats, or salt or they do contain one or more of those added ingredients but do not exceed nutrient or energy thresholds.

^3^ Not high-in beverages models do not include two-part models because the monthly percentage of consumers is >90%.

Purchase data provided by Kantar WorldPanel Chile.
