# Supplementary material for: An evaluation of Chile’s Law of Food Labeling and Advertising on sugar-sweetened beverage purchases from 2015 to 2017: A before-and-after study
Source: PLoS Med. 2020 Feb 11;17(2):e1003015. doi: 10.1371/journal.pmed.1003015 (PMC7012389; doi:10.1371/journal.pmed.1003015)
Supplement: S9 Table — (DOCX) [file pmed.1003015.s009.docx]

**S9 Table. Sensitivity analysis**

|  | **VOLUME** | | | | |
| --- | --- | --- | --- | --- | --- |
|  | **Absolute Difference** | | **Relative Difference** | |  |
|  | mL/capita/day | 95% CI | % | 95% CI |  |
| **Price adjusted^1^** |  |  |  |  |  |
| **High-in^4^** | -22.9** | (-23.0, -22.8) | -23.8% | (-23.9, -23.8) |  |
| **Not high-in^5^** | 25.3** | (25.2, 25.4) | 8.6% | (8.6, 8.6) |  |
| **Total** | -10.2** | (-10.2, -10.1) | -2.5% | (-2.5, -2.5) |  |
| **Tax period model^2^** |  |  |  |  |  |
| **High-in** | -30.0** | (-30.1, -29.9) | -29.2% | (-29.3, -29.2) |  |
| **Not high-in** | 4.4** | (4.3, 4.5) | 1.4% | (1.4, 1.5) |  |
| **Total** | -7.7** | (-7.9, -7.5) | -1.9% | (-2.0, -1.9) |  |
| **NFP linkage model^3^** |  |  |  |  |  |
| **High-in** | -16.9** | (-17.1, -16.8) | -17.4% | (-17.5, -17.3) |  |
| **Not high-in** | 7.3** | (7.2, 7.4) | 2.5% | (2.4, 2.5) |  |
| **Total** | -8.1** | (-8.1, -8.1) | -2.0% | (-2.0, -2.0) |  |

^1^ The price-adjusted model includes controls for aggregate monthly/year/region prices of beverages.

^2^ The tax period model extends the pre-period from Jan 1, 2015–June 30, 2016 to Jan 1, 2014–June 30, 2016.
It also includes a pre/post-tax dummy variable in October 2014 for the tax rate change.

^3^ The NFP linkage model uses NFP data from the pre-period (2015 and 2016) to link to purchases made from
July 1, 2016 to December 31, 2017. (The regular model uses NFP data from the post-period [2017] to link to
purchases made during this 6-month period).

^4^ High-in beverages are those subject to the Chilean Law of Labeling and Advertising due to containing added sugars, saturated fats, or salt and exceeding nutrient or energy thresholds.

^5^ Not high-in beverages are not subject to the Chilean Law of Labeling and Advertising because they either do not contain added sugars, saturated fats, or salt or they do contain one or more of those added ingredients but do not exceed nutrient or energy thresholds.

** *p* < 0.001

Purchase data provided by Kantar WorldPanel Chile.
