# Supplementary figures and images for: An evaluation of Chile’s Law of Food Labeling and Advertising on sugar-sweetened beverage purchases from 2015 to 2017: A before-and-after study
Source: PLoS Med. 2020 Feb 11;17(2):e1003015. doi: 10.1371/journal.pmed.1003015 (PMC7012389; doi:10.1371/journal.pmed.1003015)

**S1 Fig. Chilean front-of-package warning labels**

**
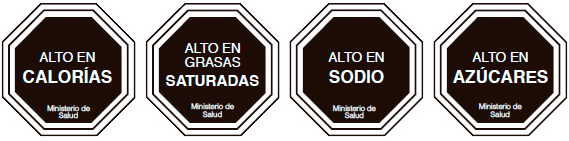
**

Supplement: S1 Fig — (DOCX) [file pmed.1003015.s010.docx]
