# Supplementary material for: An evaluation of Chile’s Law of Food Labeling and Advertising on sugar-sweetened beverage purchases from 2015 to 2017: A before-and-after study
Source: PLoS Med. 2020 Feb 11;17(2):e1003015. doi: 10.1371/journal.pmed.1003015 (PMC7012389; doi:10.1371/journal.pmed.1003015)
Supplement: S2 Fig — (DOCX) [file pmed.1003015.s011.docx]

**S2 Fig. Chilean regulation timeline and study data collection periods**

**
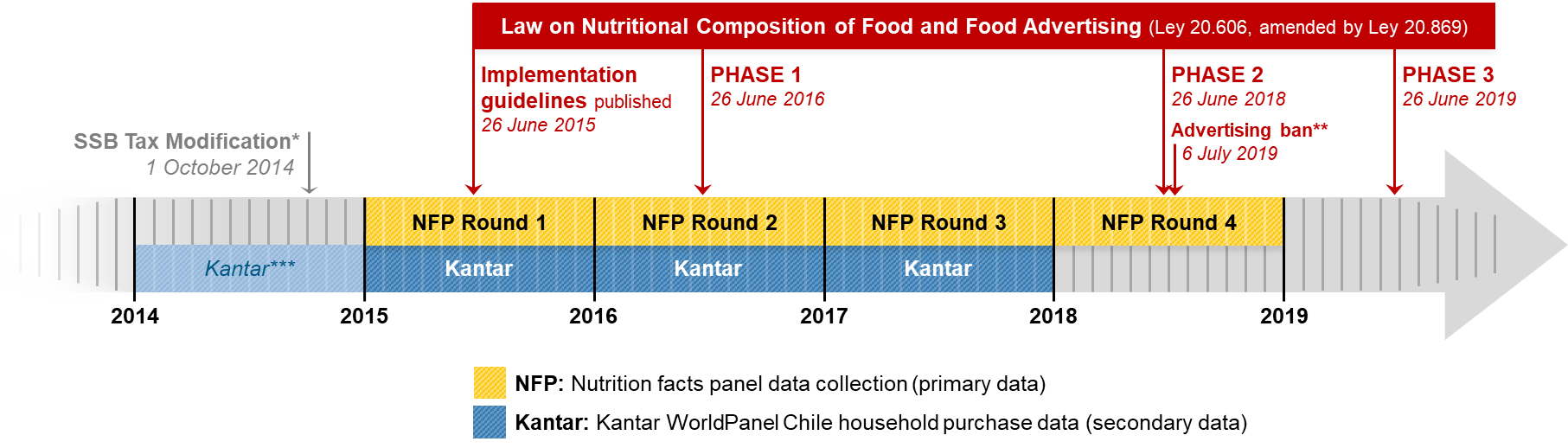
**

* SSB tax modification included an increase in tax rate from 13% to 18% for beverages containing ≥6.25 grams sugar per 100 mL
and a decrease in the tax rate from 13% to 10% for beverages containing <6.25 g sugar per 100 mL

** Advertising ban refers to the restriction of advertising on TV, radio, or cinema from 6:00 a.m.–10:00 p.m. any food or beverage product
that contains added sugars, saturated fats, or sodium and exceeds set nutrient thresholds.

*** Kantar WorldPanel Chile household purchase data for sensitivity analysis
(during SSB tax period prior to implementation of Labeling & Advertising Law
