# Supplementary material for: An evaluation of Chile’s Law of Food Labeling and Advertising on sugar-sweetened beverage purchases from 2015 to 2017: A before-and-after study
Source: PLoS Med. 2020 Feb 11;17(2):e1003015. doi: 10.1371/journal.pmed.1003015 (PMC7012389; doi:10.1371/journal.pmed.1003015)
Supplement: S3 Fig — (DOCX) [file pmed.1003015.s012.docx]

**S3 Fig. Monthly unadjusted weighted mean purchase^1^ volume
of beverages, 2015–2017**

^1^ Purchase data provided by Kantar WorldPanel Chile.

^2^ High-in beverages were those subject to the Chilean Law of Labeling and Advertising due to containing added sugars, saturated fats, or salt and exceeding nutrient or energy thresholds.

^3^ Not high-in beverages were not subject to the Chilean Law of Labeling and Advertising because they either did not contain added sugars, saturated fats, or salt or they did contain one or more of those added ingredients but did not exceed nutrient or energy thresholds.
