# Supplementary material for: An evaluation of Chile’s Law of Food Labeling and Advertising on sugar-sweetened beverage purchases from 2015 to 2017: A before-and-after study
Source: PLoS Med. 2020 Feb 11;17(2):e1003015. doi: 10.1371/journal.pmed.1003015 (PMC7012389; doi:10.1371/journal.pmed.1003015)
Supplement: S4 Fig — (DOCX) [file pmed.1003015.s013.docx]

**S13 Fig. Mean changes in purchase^1^ volume of high-in^2^ beverages, stratified by tertile of household assets index**

^1^ Purchase data provided by Kantar WorldPanel Chile.

^2^ High-in beverages were those subject to the Chilean Law of Labeling and Advertising due to containing added sugars, saturated fats, or salt and exceeding nutrient or energy thresholds.

**p* < 0.001 for the difference between observed mean absolute values and counterfactual mean absolute values in the post-regulation period.
