# Supplementary material for: An evaluation of Chile’s Law of Food Labeling and Advertising on sugar-sweetened beverage purchases from 2015 to 2017: A before-and-after study
Source: PLoS Med. 2020 Feb 11;17(2):e1003015. doi: 10.1371/journal.pmed.1003015 (PMC7012389; doi:10.1371/journal.pmed.1003015)
Supplement: Attachment — Submitted filename: taillie.pdf [file pmed.1003015.s015.pdf]

This paper discusses consumption of beverages following a policy change in Chile.

I was asked for a statistical report and I interpret that to include all aspects of the design and conduct of the study.

Points of detail

Page 7 I assume that the authors have not in fact used the two tertiles in modelling but instead used tertile categories or thirds, an ambiguity which could be removed.

Page 7 I can see why you might want to classify assets for reporting but for modelling this seems to throw away data. Categorising an essentially continuous variable wastes information (Altman and Royston, 2006; Royston et al., 2006) and leads to models which are often implausible as they predict the effect remaining flat within categories and then jumping to a new value at the category boundary.

Page 7 How exactly was household composition categorised. The categories presented here (and on page 10) seem to be neither mutually exclusive nor together exhaustive.

Page 7 I think the fact that some sub–groups were too infrequently present to be analysed is properly part of the results.

Page 9 The rather short lead in period is justified here by reference to the earlier tax change in Chile. Should this be flagged earlier (on page 4) when the time period is first introduced? Of course the sensitivity analyses belong here.

Page 11 I did not understand the parenthetical comment about the model which is not shown. I suspect showing it might help.

Page 11 The adjusted results get confidence intervals so I think the unadjusted should too.

Supplementary Tables 3 and 4 $p = 0.000$ should be $p < 0.0005$ or similar

Supplementary Table 5 What are the values in []? I would avoid asterisks as they might be interpreted as referring to significance levels. What is the suffixed + sign for?

Point of more substance

The labels constitute a four pronged attack: calories, sugar, salt, and saturated fat. The authors have analysed just beverages which are predominantly labelled for sugar. They are surprised when they put this into context with earlier Chilean studies and results from Mexico. I can see the present interest in SSB but would one way of clarifying the surprising results be to look at what the effect of the other three labels was? I can see that is a lot of extra work but at the moment this does not evaluate the whole potential public health impact of the legislative change.

Summary

Points for clarification and a query about the impact.

Michael Dewey

References

D G Altman and P Royston. The cost of dichotomising continuous variables. *British Medical Journal*, 332:1080, 2006.

P Royston, D G Altman, and W Sauerbrei. Dichotomizing continuous predictors in multiple regression: a bad idea. *Statistics in Medicine*, 25:127–141, 2006.
