# Supplementary material for: An evaluation of Chile’s Law of Food Labeling and Advertising on sugar-sweetened beverage purchases from 2015 to 2017: A before-and-after study
Source: PLoS Med. 2020 Feb 11;17(2):e1003015. doi: 10.1371/journal.pmed.1003015 (PMC7012389; doi:10.1371/journal.pmed.1003015)
Supplement: Attachment — Submitted filename: Chile SSB FOPL Eval_Plos Med Response Letter_25Oct.docx [file pmed.1003015.s016.docx]

*********************************************

Dear Dr. Misra and reviewers,

Thank you for these very thoughtful comments. We have considered these comments carefully and have responded point-by-point below. Noteworthy changes include:

- We have revised the manuscript to more fully reflect the package of policies that was implemented as part of Chile’s Labeling and Advertising Law, including the front-of-package warning labels, the marketing restrictions, and the ban of unhealthy foods and beverages in schools.
- We updated results to reflect more up-to-date educational variables provided by our data vendor, Kantar Worldpanel Chile, although results have remained consistent. We also formally tested for interaction for education and found that it was statistically significant, and have incorporated that analysis in our methods, results, and discussion.

We believe the revised paper is a stronger presentation of our findings, and we look forward to further review.

Sincerely,

Camila Corvalan

*********************************************

Requests from the editors:

-Title- please consider including " before-and -after study"

**Our response: we have included this.**

- Please consider combining first two sentences of the Abstract Methods and Findings.

**Our response: we have done this.**

-Please provide basic demographic details in the abstract

**Our response: we have done this.**

- Please provide p values with 95% CIs where appropriate

**Our response: Thank you for this suggestion. We have given this careful thought and would prefer to retain the use of confidence intervals to interpret the scientific meaning of our main results, rather than p-values, in accordance with developments in statistical thinking, much of which have been published this year. ^1-3^ Our concern with p-values, is no meaningful categorical difference between p-values of 0.051 and 0.49, and also that the use of p-values and the term “statistically significant” can lead readers (and authors) to mistakenly dichotomize results as scientifically meaningful or not, when more information about the magnitude of effect and uncertainty is needed. Instead, we prefer the approach promoted by Mathews as well as that which was promoted in a recent Nature commentary, in which the focus is more on “compatibility intervals” or “analysis credibility” using confidence intervals. The advantage to confidence intervals is that they provide more information: their bounds give insight into the magnitude of the effect, along with the direction, but also provide information about the associated uncertainty. To clarify our approach, we have added a note to the methods section regarding our use of confidence intervals, as well as the relevant citations. In cases where a p-value was requested specifically by reviewers (for example, the p-value of the interaction term for education and time in our educational analyses), we have added this.**

- At this stage, we ask that you include a short, non-technical Author Summary of your research to make findings accessible to a wide audience that includes both scientists and non-scientists. The Author Summary should immediately follow the Abstract in your revised manuscript. This text is subject to editorial change and should be distinct from the scientific abstract. Please see our author guidelines for more information: <https://journals.plos.org/plosmedicine/s/revising-your-manuscript#loc-author-summary>

**Our response: we have done this.**

- Please provide a completed STROBE checklist to comply with our reporting requirements. Please ensure that you don't use page numbers as these are likely to change

**Our response: We have uploaded the STROBE checklist with out revised materials.**

- as noted by reviewers, a reference listed as under review. Please note this is not permitted as per PLOS Data policy. We would recommend providing further details regarding this reference

**Our response: This paper has since been published and we have updated the reference. (Note we do have one “in press” citation that we anticipate will be published within the next month; we will remove it if it is not formally published online prior to future revision or publication of this article.)**

-Please state "study limitations include...." or something similar around line 411

**Our response: we have included this.**

Editorial notes:

1) To enhance the reproducibility of your results, we recommend that you deposit your laboratory protocols in protocols.io, where a protocol can be assigned its own identifier (DOI) such that it can be cited independently in the future. For instructions see <http://journals.plos.org/plosmedicine/s/submission-guidelines#loc-methods>.

**Our response: We have done this and mentioned and cited it in the Methods (Line 127).**

In addition, we request that you upload any figures associated with your paper as individual TIF or EPS files with 300dpi resolution at resubmission; please read our figure guidelines for more information on our requirements: <http://journals.plos.org/plosmedicine/s/figures>. While revising your submission, please upload your figure files to the PACE digital diagnostic tool, <https://pacev2.apexcovantage.com/>. PACE helps ensure that figures meet PLOS requirements. To use PACE, you must first register as a user. Then, login and navigate to the UPLOAD tab, where you will find detailed instructions on how to use the tool. If you encounter any issues or have any questions when using PACE, please email us at [PLOSMedicine@plos.org](mailto:PLOSMedicine@plos.org).

**Our response: We have done this.**

Comments from the reviewers:

Reviewer #1: The stated purpose of this manuscript is to evaluate the impact of Chile's labeling and advertising law on household beverage purchases. One major concern is that the policy(s) of interest change throughout the manuscript and co-occurring policies/practices in Chile make it impossible to tease out the impact of the front-of-package warning label on household purchases of SSBs. With respect to the changing policy of focus for the evaluation, the paper title and abstract point only to the association between the Chilean front-of-pack labeling policy and household SSB purchases while the intro and discussion point to the association between both the Chilean labeling and marketing regulations and household SSB purchases. With respect to co-occurring policies/practices, the introduction also mentions a third policy in Chile - restrictions on the promotion and sales of certain foods and beverages in schools which went into effect at the same time as the marketing

regulation (June 2016). Based on the study they cite, it is probable that this school policy may also have effected household SSB purchases. In addition, the discussion mentions a mass media campaign by the Ministry of Heath to inform customers about the meaning of the warning labels and encouraging them to choose products with fewer labels. The combination of the observational study design and co-occurring policies/practices makes it very hard (if not impossible) to interpret the observed decline in SSB purchases. And the fact that the authors observe a much bigger impact than a recent meta analyses on food labeling (although it was not just FOP) suggests this is not a pure FOP effect (which the authors acknowledge may be the case).

To address the two concerns described above, the paper should be recast to clearly focus on all the co-occurring policies/practices in Chile, not just FOP. And since the UNC team has considerable data from various countries in South America, it would be ideal to include data from a comparable control country without the same suite of policies as Chile and conduct a natural experiment with a difference-in-differences approach. This would be much more robust analytical approach, and while it could not tease apart the impact of the various policies/practices on household SSB purchases, it could answer the question of whether the observed changes in household SSB purchases in Chile are due to the combination of policies/practices or just secular trends in the region.

**Our response: We appreciate this very wise comment. First, we fully agree about reframing the paper to acknowledge the full set of policies, and we have updated the title, abstract, introduction, and discussion accordingly. We have also changed how we refer to the beverages that were regulated. Instead of calling these warning-labeled beverages, we now take the emphasis off the labeling and instead call them “high-in” beverages (because they are high in nutrients of concern, according to the nutrient thresholds established by the Chilean regulation).**

**Secondly, we agree that a cross-country comparison would be very useful for the reasons mentioned. Unfortunately, we do not currently possess cleaned, organized data for a comparable country during the similar time period. However, we agree that this approach of using another country as a control is very important and we intend to address this in future work. We have added a comment on this in the limitations and note it as a direction for future research.**

Another major concern is the time frame of the pre and post data. The pre period is from January 2015 to June of 2016 and the post period is from July 2016 to December 2017. While the authors do adjust for month to account for seasonality, the pre and post periods are not overlapping in terms of calendar months. This is a big issue since lots of literature has shown that people purchase beverages differently depending on the time of year. And beverage purchases may be particularly sensitive to weather, month, holidays etc. Ideally, the authors should have one full year prior and one full year post. If that is not possible, the authors should use the same months for the post period (January 2016 to June of 2017). Without making this change, it is not possible to interpret the observed decline.

**Our response: We believe that our approach of using 18 months before and after is a better alternative for the following reasons. First, we have balanced pre- and post- periods: 18 months prior to the regulation implementation and 18 months after the regulation implementation period. There is overlap in the months: both periods contain a January to December period. The difference is that the pre-period contains an additional 6-month period in the first semester (January to June, 2016) and the post-period contains an additional 6-month period in the second semester (July to December, 2016). Secondly, the longer we go before the regulation was implemented, the better, because we adjust for potential pre-regulation trends. Finally, we are adjusting for month of the year to adjust for seasonality (dummies for month). Still, we ran the model with different window time periods to see how robust our results were and found that in general. However, when cut down to 12 months before and after, we found that the results are sensitive to how seasonality is specified. When including month as a continuous variable, the results are very similar to the 18 months (our specification with either with month as dummies as presented in the paper or as a continuous variable). However, when we adjust for seasonality in the 12 months before and after, using quarters to control for seasonality, we get very large negative results but when we include dummies for month to control for seasonality, we get very large positive results. This is in contrast to the 18 month period. We conducted the same set of analyses using the 18 month period (using quarters, using month as a continuous variable, and using month as dummies), and found that the results are no longer sensitive to how seasonality is specified (i.e., we get very similar results using the different approaches). Because of the reasons mentioned above and because the 18 month period is robust to specifications for seasonality, we concluded that a longer period is needed so we decided to leave the results with 18 months before and after.**

A more minor point is that since their Kantar panel only includes store bought beverages and not purchases outside the home such as at restaurants or street vendors, it would be helpful to add a sentence to the introduction about what fraction of beverage sales are store bought. If it is small, that should be included as a limitation.

**Our response: Thanks for this important point. To the introduction, we have added a note (Line 119) that we are studying beverage purchases made from stores. From Euromonitor sales data, we found that 88% of beverages are store-bought, and this figure remained constant over time. We’ve added this information in the discussion (limitations; Line 554). We also note that this is an important reason why it will be necessary to evaluate changes in dietary intake, in order to see how this regulation affected consumption across the range of sources (e.g., if consumers simply shift from purchasing store-bought drinks to restaurant drinks, the regulation may not be impactful from a biological perspective).**

Another minor point is that the authors cite a paper under review. I would remove this cite and the associated evidence until it is in press.

**Our response: We agree and have removed this under review reference. All references have been published, except one “in press” citation that we anticipate will be published within the next month; we will remove it if it is not formally published online prior to future revision or publication of this article.**

Reviewer #2: This is a good attempt to evaluate an complex, but internationally important, public health intervention. Further justification of the approach taken and clearer conceptualisation of the intervention and its potential impacts would be valuable.

Substantive conceptual comments

The conceptualisation of exactly what the intervention of interest is is inconsistent. Only warning labels are mentioned in the title and abstract. Concurrent marketing restrictions are mentioned in the introduction. A concurrent mass media education campaign is mentioned in the discussion. In some places the results are attributed simply to the warning labels, in other places the marketing restrictions are also recognised as playing some role, in one place it's acknowledged that it's impossible to "differentiate effects" of the intervention components. I think it would be more honest to provide a much clearer and consistent conceptualisation of the intervention as the combination of the simultaneous introduction of warning labels and marketing restrictions alongside mass media education. This will require some re-pitching of the introduction; refinement of the text throughout; and further consideration of what the relevant comparator literature is in the discussion. It is

not surprising to me that a multi-component strategy introduced in the context of a pre-existing SSB tax has a greater effect than a tax alone - and the need for multi-component strategies, rather than single 'magic bullet' interventions, is an incredibly important policy message.

**Our response: We fully agree and have reshaped the title, abstract, intro, tables/figures, and discussion to reflect this important point. We’ve also added specific sentences in the abstract, author summary, and conclusion to draw attention to the fact that this is a package of policies, not just a single policy. We have also changed what we call the beverages to take the focus off labeling; that is, we now refer to beverages that are regulated as “high-in” (because they are high in nutrients of concern, according to the Chilean regulation), rather than “warning-labeled).**

The rationale for focusing on beverage purchasing specifically, rather than purchasing of all labelled food, is not clear to me. Whilst SSB consumption has clearly been a particular area of recent policy focus, I'm not sure we should uncritically privilege it as the main public health problem of our time. Perhaps some evidence to indicate the PAF of eg obesity or diabetes for SSBs might provide some justification? Do you have comparable data for food that could be included? If so, I'd prefer to see that here so we can get a more comprehensive picture.

**Our response: We have included additional rationale for the focus on SSBs (particularly the biological mechanism and why they are uniquely harmful, compared to foods) in the introduction in Line 68. We agree data on food would be very useful; however, because foods are much more heterogenous and more numerous than beverages, that data was not yet fully cleaned, categorized, and linked to nutrition facts panel data at the time of this publication as this is a years-long process to ensure accuracy of the nutritional profile of foods during a period of rapid reformulation. We do think that there have been sufficient policies focused specifically on SSBs to warrant that focus here (>40 countries with SSB taxes). However, we do note it will be important to see the effects across total food purchases as well as effects on total dietary patterns; we have added this to the discussion section (limitations section, lines 570–575).**

An implicit rationale for focusing on beverage consumption appears to be the ability to contrast effects of taxes against the current suite of interventions (as per Fig 3). I think this is an unfair comparison in this context. The current interventions were introduced in the context of an existing tax - this is not an either/or situation. It is entirely feasible that the interventions studied here would not have had the same effect without the tax also being in place**.**

**Our response: While it is true that we are not entirely able to distinguish the effects of the existing set of Chilean regulations from their previous SSB tax modification that occurred 20 months prior, our previously published paper has found that Chile’s SSB tax modification was quite small (from 13% to 18% for high-sugar drinks and 13% to 10% for low-sugar drinks and was part of a major overall tax reform) and had a very small effect on prices and purchases (3%). Given that we controlled for pre-existing trends, and conducted a sensitivity analyses which included the tax period and additional dummy for the tax, we feel that we have been able to the extent possible to isolate the SSB tax. Moreover, the impact of taxes on purchases occurs when consumers respond to change in price (increase). Given that the tax was implemented over a year and a half before the labeling and advertising regulation, there is no reason to expect there was a price increase that occurred simultaneously with the labeling/advertising law. Nevertheless, we have added a note to the limitations to say note that other changes in industry behavior, such as price changes, need to be explored in further detail.**

**With regards to comparison to other taxes, we felt this comparison was essential to include because we believe it will be useful for scientists, advocates, and policymakers who are selecting from an array of potential policies to reduce SSBs or other foods. However, we agree that the stronger point is that a package of policies (Chile’s labeling, marketing and school policies) is stronger than a single policy (tax) and so we have added a footnote the figure to correct this and amended the title. We also added to the discussion several points discussing the fact that this is a package of policies and that is likely why we see the bigger changes for this vs. the standalone policies like SSB taxes or labeling-only policies (paragraph beginning line 453, paragraph beginning line 470).**

The exclusion of out-of-home purchases seems a major limitation that little attention is paid to. Are there other sources of data that you could use to study this? Can you say a little more in the discussion about what biases this might impose?

**Our response: This is a wise point and a Kantar limitation that we cannot measure away-from-home eating. Only Euromonitor allows this measurement at the national level. While the vast majority of purchases are made in stores (88%, we have added a note on this to the limitations, paragraph starting 547, particularly the need to look at purchases and intake across sources).**

I'm not sure why average purchases across the whole post intervention period vs counterfactual is the most appropriate way of expressing impact. I would have thought this mean is heavily influence by the length of time for which post-intervention data available. I wonder if a more appropriate approach would be to choose a policy relevant 'endpoint' for the study and estimate change between observed and counterfactual at that point. Perhaps immediately prior to implementation of next regulations would be sensible - which is the most sustained impact of the current intervention that can be measured before an additional intervention is added.

**Our response: First, we believe looking at the average across the time period is most useful for understanding the impact on public health, because it represents changes in purchasing patterns over time, which is important for understanding whether we are likely to see sustained changes in dietary intake. If we choose a policy “endpoint”, results may be biased if this was a small and unusual period. Taking a longer period of time allow us to adjust for pre-regulation trends. In addition, a year and a half is a relatively short period post-regulation. Unfortunately, we do not have data all the way until the implementation of the next set of regulations (our data ends in December of 2017; the next regulation began in late June of 2018). We do agree that understanding the long-term effects of this regulation will be very important (both as the regulation gets stricter over time as well as to understand whether there are any “wear out” effects); we have added this to the discussion (paragraph beginning with Line 531).**

Other comments

The manuscript includes numerous typos, repeated words, missing 'minus' signs, trailing phrases, and even one "ref" where a reference seems yet to be added.

**Our response: Thanks for catching this. We’ve fixed the missing reference and had a proofreader check for typos, etc.**

I'm not sure that "fiscal policies have been the major approach for reducing [SSB] intake". They have been a big focus recently. But prior to that there were e.g. educational campaigns related to dental health and sugary drink consumption; and even now I think governments might say they are doing quite a lot to reduce consumption that isn't taxes - education, labelling, supporting reformulation, encouraging reduce package size etc.

**Our response: We have added the word “recent” to clarify that the emphasis on taxes has been recent, with most efforts implemented since 2014. In fact now over 42 countries and US cities have instituted SSB taxes in this decade and more are under final consideration.**

I'm also not sure that "front of package warning labels, marketing restrictions, and bans" have been "more recently" discussed. I think these have been considered at least as long as fiscal policies.

**Our response: We have reframed this so that this paragraph is less about “recent” discussions.**

Whilst I agree that "education may influence purchasing decisions by affecting how well an individual is able to understand the information communicated in nutrition messages", I think a major additional issue here is that education is likely to be associated with greater (financial and non-financial) resources that allow individuals to enact the changes encouraged.

**Our response: We concur that education cannot be fully separated from its correlation with financial and non-financial resources. This is an important point and we have added it along with a reference (Line 114).**

If I understand supplemental fig 2 correctly, the guidance on the intervention assessed here was published in June 2015 - six months after the pre-data phase began. It seems possible that this publication represented a further 'intervention' with the potential to change both industry and consumer behaviour. From the authors knowledge of the context, is this feasible? If so, how does the presence of this additional intervention in the pre-period impact interpretation?

**Our response: We do not think it likely that this 2015 announcement had an impact. First, unlike Mexico and many other countries, there was no major media and advocacy period leading up to the regulations, so there is no reason to think that this guidance would have changed consumer behavior. In addition, our group’s previous study on the anticipatory effects of the law found that virtually no reformulation of products occurred between 2015 and 2016 prior to implementation, suggesting that there were not major changes in the food supply leading up to the law. ^4^ Finally, even if there hypothetically was some impact, it seems feasible that this would have led to a decrease in SSB purchases during the pre-regulation period, reducing the difference we would see between what we observed in the post-period and what as expected in the post-period based on the pre-period trend. Thus, in the scenario where there was some anticipatory effect, our results err on the more conservative side.**

I don't understand why "a team of Spanish-speaking nutritionist research assistants" were needed to code products as label-able or not. Wasn't this an automated calculation done using NFP data?

**Our response: We wish it were this simple. We use a computer algorithms to make an initial match, but because the nutritional thresholds for the Chilean law are complex, it is essential that a nutritionist review each product to ensure accuracy. To provide more detail: the Kantar data is linked to NFP data at the product level. We do this programmatically. However, not all products have a direct match. In cases where products do not have a direct match, imputation needs to be done to the nearest flavor, brand, or category, depending on what is available. This requires someone with a knowledge of the Chilean food supply, ability to read labels, and a knowledge of what is most nutritionally relevant. Second, the NFP that is collected and entered is not always entirely accurate or has data missing, due to what is present (or absent) on the label itself. While we can flag outliers and missing data programmatically, again, they must be corrected using an imputation approach that requires advanced nutrition knowledge. For example, if a product reports 0 calories but 1 gram of sugar, we need to determine whether it ought to be the calories that should be corrected, the sugar value that should be corrected, or if the product should remain as is because this is the best representation of what is publicly available knowledge about the product.**

**Of equal importance is the issue of regulation status. Because the Chilean law is based on nutritional thresholds for foods and drinks in their “as-consumed” form, beverages such as powders and concentrates have to be reconstituted to determine their regulation status. This requires skill and reading of the reconstitution or cooking instructions on each packaged product that was powdered or in concentrated form.**

It would be helpful to label the components of the analysis (descriptive/unadjusted, fixed effects/adjusted) similarly in the methods and results to allow readers to easily join the two up.

**Our response: We agree this would be helpful and have done so. (See lines 218 and 225 in Methods and lines 291 and 313 in Results.)**

If I understand correctly, your data is per household per week, yet the results refer to per capita per day. It would be helpful to know exactly how you get from one to another and any limitations of this (e.g. is data on household size accurate and is it fair to assume equal distribution of purchases).

**Our response: Although the enumerators visit participants each week, data is recorded at the daily level. However, we aggregate this data to the monthly level for analysis. The main reason for this is because the size of the data set and the number of days is overwhelming for processing if purchases are kept dis-aggregated at the daily level. In addition, analysis at the monthly level allows us to minimize the effect of non-consumers and better represents usual purchasing pattern. We know from many food studies that obtaining a longer period for reporting food purchases or any measure of eating behavior is more likely to approach usual intake (i.e. looking at months rather than days). Purchases of packaged food and beverages are particularly lumpy so we aggregate them across a longer time period. We then placed our results into daily per capita terms for ease of interpretation (since most dietary recommendations are at the daily level) and comparability with previous studies.^5,6^**

The differential results by education appear to be descriptive only. I notice that confidence intervals don't overlap, but wondered if a formal test for interaction would be valuable.

**Our response: We used stratified models to obtain the differences between observed and counterfactual for each educational level because we were unable to apply Stata’s margins command to obtain these in a fully interacted model. However, we have included a fully interacted model to test for an interaction by education, which was statistically significant. We have included the following amendments to the manuscript:**

**Methods (paragraph starting Line 250): *To test for differences in education on changes in purchases of high-in beverages after the regulation was implemented, we ran a fully interacted model including interactions of all variables in the model with education. We then used an F-test to determine whether the triple interaction of education (specified as dummies), the regulation time period (a dummy variable for pre/post), and time (continuous) was statistically significant.***

**Results (Line 322): *In the fully interacted model (results not shown), the interaction between education, regulation period, and time was statistically significant (p<0.001).***

Two evaluations of the impact of the Chile SSB tax were published simultaneously. However, only one is referred to in the comparison of effects.

**Our response: This is correct. We believe that the other evaluation of the SSB tax is highly erroneous considering that they found a change of 20% decline in purchases on a very small (3%) increase in price, a finding which defies what would be expected based on -1.3 price elasticity on SSBs.^7^ We have spoken with the authors of this paper and been unable to determine the reason behind the discrepancy in findings. However, to be fair, we have added a note about this comparison in our discussion (Line 461).**

**We want to add a separate point. A group of eminent economists were going to publish a critique of that other SSB tax impact paper but our Chilean collaborators asked that this not be published since they were colleagues in the same university. We were surprised by the publication simultaneously of the two differing papers without a commentary but a coauthor of the paper who is a Plos Med editor stayed out of any interaction based on a conflict of interest.**

I think the asterisks in supplemental table 5 title and table body may refer to different things, but the footnotes don't make this clear. Also not clear what is presented in square brackets in this table. Please check through all tables for similar problems.

**Our response: The new table specifies the brackets are for standard errors and asterisks are for p-values.**

In addition to the unadjusted trend plots in supplemental fig 3, I would like to see conventional ITS figures plotting adjusted pre and post trends alongside post counterfactual. This would allow readers to helpfully visualize e.g. acceleration of pre-existing trends.

**Our response: We adapted an ITS approach to a panel, so we did not run a traditional ITS that is based on time series, which is why we do not present these figures. Information on pre-regulation trends is available in the supplement (Supplemental Figure 3, coefficients in Supplemental Table 5).**

I think it is incorrect to conclude that "similar absolute changes by household education suggests that this regulation is not likely to increase disparities". If inequalities are measured on a relative scale then similar absolute reductions can result in increased relative differences.

**Our response: Thank you for this very wise point. We have updated our discussion to take a more nuanced approach and point out that it could be problematic if this law leads to increased relative differences between high vs. low education (Lines 448–449).**

It's mentioned in the discussion that you cannot disentangle consumer vs industry changes. However, I think from the NFP data you have, you could provide some insight into the extent of reformulation. Could that be included here?

**Our response: There are two issues. We are working on some complex econometric methods that would allow us later to separate supply and demand affects. Even reformulation and new product introduction reflects an industry response but is impacted by consumer demand. At the same time, discussing reformulation is very complex if you separate product changes from new product introduction. We have a separate paper on reformulation that is currently under review and we believe important enough to merit its own paper. We cannot cite the paper as it is under review but do note in the discussion that our results on policy impact are likely a combination of changes by the industry via reformulation and new product introduction as well as consumer demand changes.**

I don't see how the interpretation that the "policies accelerated pre-existing trends" is "another interpretation". Surely the only interpretation is there were pre-existing trends, they were steeper post intervention.

**Our response: We agree and have deleted this paragraph (also for space).**

Reviewer #3: This is an important study that evaluates the effect of a multitude of labeling and marketing policies on beverage purchases in Chile. These policies are unique and were the first of their kind to be implemented. The dataset used seems comprehensive and adequate for the this evaluation, and the methods used are the ideal for this type of analysis. Authors explored an extensive range of sensitivity analyses to determine if results were robust to different model specifications and data manipulations, and they preregistered their analytic plan. Weaknesses are clearly articulated and common with this type of observational research. Few suggestions and questions:

1) The authors cite a publication that further describes the database used for their evaluation. It might be helpful to get some basic information on that source into this manuscript (understanding word limitations are a challenge). For example, it would be helpful to know the survey response rate and whether there were specific restrictions/exclusions for data collection.

**Our response:** **The response rate for the survey is 95%. Households are excluded if any member is engaged in activities determined to have potential to interfere with the collection of information about the products studied (e.g., a household member works at an advertising agency, market research company, written or spoken media company, or is owner of company that markets a product studied). Households are also excluded if they do not meet minimum purchasing standards (e.g., purchase at least one of item from one of the 15 categories from the “basic basket” of goods). We have added this information starting in Line 131.**

2) Was any information on policy compliance available from the data collectors who evaluated products in homes? I would imagine that some of the products that met warning label thresholds weren't labeled, and these products might have systematically differed from those that were labeled. This should have biased results to the null but would be interesting to see nonetheless, if it was available.

**Our response: Unfortunately, policy compliance was not evaluated by the data collectors from Kantar WorldPanel. We are planning to look at compliance, but since this information is at the product level, we are including it in a separate manuscript on reformulation of products and compliance (currently under review).**

3) In an interrupted time series analysis, investigators examine the post-intervention level change as well as the trend change (effect size from the indicator of prev. post as well as the interaction term with time). Those results seem to be in Supp Table 5, and if my interpretation is correct, there is a pre-post level change but no real trend difference pre vs. post. Is that correct? Could be highlighted in manuscript, esp bc suggests no clear attenuation of effect over time (though still only a short period of post-implementation data).

**Our response: We agree with the reviewer, this is our interpretation. We found a significant change in purchases immediately after the regulation was implemented but not a change in the post regulation trend, suggesting the effects did not change (attenuate or increase). We also believe that could be due to a short period of post-implementation data, and these results may change as the novelty wears off or conversely, if social norms shift to further reduce preferences for high-in beverages. We added this to the discussion (Line 534).**

4) Authors display in Table 1 that the survey responders were different pre vs. post. Any sense of why this might have happened? Stratifying the results by education and assets, as the authors do, certainly helps this, but it does seem peculiar to have such an increase in the education level post-regulation. Was there a requirement, for this study, that panel members had some data in the pre and post period, or was all data included regardless of whether participants were in both periods?

**Our response: We also noticed this and investigated with our data vendor, Kantar WorldPanel. It took a long time to work out with them, but it turns out that they had provided mis-coded education for some households for the years 2016 and 2017. These have been corrected in all analyses and new results on education are presented. This correction did not affect our overall results.**

5) This is really a style preference, but some of the top line results presented are relative instead of absolute changes. I think the absolute changes might be more helpful (might just require switching out the figures and presentation of result to show the absolute differences as the main outcome). The comparison with other policy effects is very useful and puts the small changes here into context - small but important at the population level and larger than what was seen from the SSB tax in Mexico.

**Our response: In most cases, we present both absolute and relative declines. We have adjusted the text to report absolute results followed by relative results. In the case of the figures, since the main bars show the relative results, we tried to adjust this by moving the text inside of the bar and better highlighting the absolute results.**

6) The differences reported are at the population level. This obviously includes some households that had no purchasing of labeled beverages. Would be helpful to also see what the changes were in households that had pre-implementation purchases of labeled beverages

**Our response: Because the proportion of households purchasing labeled beverages was so high (93% of households prior to implementation), we do not expect to see differences when looking at consumers-only. However, we do think it will be important to understand differences between high vs. low consumers, since much of the public health benefit could come from reducing consumption among the top consumers and have noted this in the discussion (Lines 449–452).**

7) Supplemental Figure 3 shows an uncharacteristic spike in ml purchased in Jan 2017. Any thoughts about what that represents? Could be a New Year's effect but don't really see in prior years, during the pre-reg period.

**Our response: We have investigated this issue with our data vendor. We found that they have miscoded some beverage purchases from January of a previous year (outside of our data sample) as occurring in January 2017. They have since told us that those data belong in February of 2017, which essentially just moved the spike from January to February. To better understand this issue, we tested the results with the data included as January 2017, February 2017, and excluded altogether and found that the results remain nearly identical. We believe that including the data in 2017 in general is a more conservative approach, since including this data increases the amount of “high-in” beverages purchases after the regulation, as opposed to decreasing the amount of high-in beverage purchases after the regulation as we might expect. Of course, it also increases the amount of not-high in purchases that were purchased in the post-regulation period, but we are less concerned about this, since our primary interest is in the high-in beverages. Thus, because including the data in 2017 is more conservative, and February 2017 is our best understanding of the when this data was collected according to the vendor, we decided to include the data as Feb 2017.**

8) The absolute changes in % of consumers of fruit drinks and dairy pre vs. post are stunning. Could be highlighted in the manuscript more.

**Our response: We agree, and have added these results to our top-level summary in the introduction of the discussion (Line 433).**

Jason Block

Reviewer #4: See attachment listed out below

Michael Dewey

Taillie et al September 6, 2019

This paper discusses consumption of beverages following a policy change in Chile.

I was asked for a statistical report and I interpret that to include all aspects of the design and conduct of the study.

Points of detail

Page 7 I assume that the authors have not in fact used the two tertiles in modelling but instead used tertile categories or thirds, an ambiguity which could be removed.

**Our response: This is correct. In our main models, we now include assets as a continuous control variable, per your suggestion. In order to look at the differences between high vs. low assets, as reported in the sensitivity analysis, we kept the categories based on tertiles but now explain this more clearly (Line 206).**

Page 7 I can see why you might want to classify assets for reporting but for modelling this seems to throw away data. Categorising an essentially continuous variable wastes information (Altman and Royston, 2006;Royston et al., 2006) and leads to models which are often implausible as they predict the effect remaining at within categories and then jumping to a new value at the category boundary.

**Our response: We have re-analyzed the models with assets score as a continuous variable. However, we retained the categorized version of this variable to be able to conduct the stratified analysis to examine high vs. low assets.**

Page 7 How exactly was household composition categorised. The categories presented here (and on page 10) seem to be neither mutually exclusive nor together exhaustive.

**Our response: We have updated our explanation to be more clear. The variables included in the model represent the number of people in each of the following categories: children 0-1y, children 2-5y, children 6-13y, males 14-18y, females 14-18 y, females >18 and males >18. We’ve added a more thorough explanation of this to the methods section [Lines 208–210].**

Page 7 I think the fact that some subgroups were too infrequently present to be analysed is properly part of the results.

**Our response: We have moved this sentence to the Results section [Lines 308–311].**

Page 9 The rather short lead in period is justified here by reference to the earlier tax change in Chile. Should this be flagged earlier (on page 4) when the time period is _first introduced? Of course the sensitivity analyses belong here.

**Our response: Information about the SSB tax modification is introduced in the introduction [Line 92].**

Page 11 I did not understand the parenthetical comment about the model which is not shown. I suspect showing it might help.

**Our response: The main goal of this analysis was to show pre-regulation trends in high-in beverage purchases. We have since realized that this analysis and results were redundant with one of our sensitivity analyses, which shows the co-efficient of the pre-regulation trend. Thus, we have removed this analysis and results for clarity and confusion. The co-efficients can be found in Supplemental Table 5.**

Page 11 The adjusted results get confidence intervals so I think the unadjusted should too.

**Our response: We have added these.**

Supplementary Tables 3 and 4 p = 0:000 should be p < 0:0005 or similar

**Our response: We have corrected this throughout all tables.**

Supplementary Table 5 What are the values in []? I would avoid asterisks as they might be interpreted as referring to significance levels. What is the suffixed + sign for?

**Our response: We have corrected this as well as changed the footnotes. We added asterisks for p values and note that the asterisks are for SEs.**

Point of more substance

The labels constitute a four pronged attack: calories, sugar, salt, and saturated fat. The authors have analysed just beverages which are predominantly labelled for sugar. They are surprised when they put this into context with earlier Chilean studies and results from Mexico. I can see the present interest in SSB but would one way of clarifying the surprising results be to look at

what the effect of the other three labels was? I can see that is a lot of extra work but at the moment this does not evaluate the whole potential public health impact of the legislative change.

**Our response: This is a wise point. The results actually do include the effect of all 4 types of labels, not just sugar (i.e., a beverage is considered labeled if it contained added sugar, added sodium, or saturated fat and exceeded nutrient thresholds for sugar, sodium, saturated fat, and sodium). Of course, in the case of beverages, nearly all of the beverages were labeled due to sugar, simply because that is the most common high-in nutrient and so few with two labels that such an analysis would not be possible for beverages.**

**Because we have re-framed the whole paper to make clear we are looking before/after the package of regulations, hopefully it is also more clear that the results are not so dependent on the labels in general and labels for specific nutrients in particular. However, we have also added a note to clarify that our approach looks at all high-in beverages (Line 181). We also now note in the limitations that future research should explore whether there are differences between high-in nutrients and label numbers/types (though we feel this approach will be more relevant for foods where there is more heterogeneity in the types of nutrients that appear on the warnings) (paragraph beginning Line 542).**

Summary

Points for clarification and a query about the impact.

Michael Dewey

References

D G Altman and P Royston. The cost of dichotomising continuous variables.

British Medical Journal, 332:1080, 2006.

P Royston, D G Altman, andWSauerbrei. Dichotomizing continuous predictors in multiple regression: a bad idea. Statistics in Medicine, 25:127{141,2006.

Page 2

Any attachments provided with reviews can be seen via the following link:

<https://www.editorialmanager.com/pmedicine/l.asp?i=495644&l=GQRX13R2>

__________________________________________________

In compliance with data protection regulations, you may request that we remove your personal registration details at any time. (Use the following URL: <https://www.editorialmanager.com/pmedicine/login.asp?a=r>). Please contact the publication office if you have any questions.

1.	Wasserstein RL, Schirm AL, Lazar NA. Moving to a world beyond “p< 0.05”. Taylor & Francis; 2019.

2.	Amrhein V, Greenland S, McShane B. Scientists rise up against statistical significance. Nature Publishing Group; 2019.

3.	Matthews RA. Moving Towards the Post p< 0.05 Era via the Analysis of Credibility. *The American Statistician.* 2019;73(sup1):202-212.

4.	Kanter R, Reyes M, Vandevijvere S, Swinburn B, Corvalán C. Anticipatory effects of the implementation of the Chilean Law of Food Labeling and Advertising on food and beverage product reformulation. *Obesity Reviews.* 2019.

5.	Colchero A, Rivera JA, Popkin BM, Ng SW. Beverage purchases from stores in Mexico under the excise tax on sugar sweetened beverages: observational study. *BMJ.* 2016;352(h6704).

6.	Colchero MA, Rivera-Dommarco J, Popkin B, Ng SW. In Mexico, Evidence of Sustained Consumer Response Two Years after Implementing a Sugar-Sweetened Beverage Tax. *Health Affairs.* 2017;36(3):564-571.

7.	Caro JC, Ng SW, Taillie LS, Popkin BM. Designing a tax to discourage unhealthy food and beverage purchases: The case of Chile. *Food Policy.* 2017;71(Supplement C):86-100.
